# Responsibility in healthcare across time and agents

**DOI:** 10.1136/medethics-2019-105382

**Published:** 2019-06-20

**Authors:** Rebecca C H Brown, Julian Savulescu

**Affiliations:** 1 Oxford Uehiro Centre for Practical Ethics, University of Oxford, Oxford, UK; 2 Oxford Uehiro Centre for Practical Ethics, University of Oxford, Oxford, UK

**Keywords:** public health ethics, health promotion, social aspects, philosophical ethics, ethics

## Abstract

It is unclear whether someone’s responsibility for developing a disease or maintaining his or her health should affect what healthcare he or she receives. While this dispute continues, we suggest that, *if* responsibility is to play a role in healthcare, the concept must be rethought in order to reflect the sense in which many health-related behaviours occur repeatedly over time and are the product of more than one agent. Most philosophical accounts of responsibility are synchronic and individualistic; we indicate here what paying more attention to the diachronic and dyadic aspects of responsibility might involve and what implications this could have for assessments of responsibility for health-related behaviour.

## Introduction

The appropriate role for personal responsibility in healthcare continues to be debated.^1–6^ While some think that healthcare provision ought to be blind to considerations of responsibility and desert, others suggest that it is appropriate to take account of responsibility in making decisions about healthcare. We are sympathetic to arguments that responsibility should not play a role in healthcare. However, we also recognise that responsibility practices are a commonplace feature of almost all areas of human life and interpersonal relationships. Given that the question of *whether* responsibility should play a formal role in healthcare remains unsettled, and furthermore that judgements about responsibility are likely to motivate at least some policies and practices in healthcare, we aim to consider how theories of responsibility could be usefully adapted to provide insight into people’s responsibility for their health.

We argue that approaches to thinking about responsibility for health could usefully be enhanced by sufficiently acknowledging two key factors. First, that health-related behaviour is often the product of multiple choices and actions over time, and second, the extent to which a given person’s health behaviour is the product of both her own and close others’ (dyadic) actions. We suggest that properly acknowledging these diachronic and dyadic aspects of responsibility is important when considering people’s responsibility for their health, particularly relating to habitual behaviours and management of long term/chronic conditions. We do not claim that judgements of responsibility based on this fuller analysis would justify particular healthcare policies or responses to particular individuals. Such questions demand further interrogation.

We begin by outlining some of the ways in which responsibility is used in healthcare policy and practice to illustrate the pervasiveness of this concept and the improbability of banishing it entirely. We then discuss how current uses of responsibility pay insufficient attention to the ways in which agents’ responsibility for actions can be judged at different time points and the ways in which responsibility for actions can be distributed across different agents. We describe how responsibility can be both *diachronic* and *dyadic*, particularly in the context of health-related behaviour and discuss how attending to these dimensions could have implications for healthcare.

## Responsibility in healthcare

Moral responsibility is a commonplace feature of human societies. Responsibility practices regulate personal relationships, uphold criminal justice, incentivise productive work and discourage antisocial behaviour. Allocation of responsibility may well be an inevitable result of the way human psychology and social interactions are structured. As Strawson^7^ argues, attributions of responsibility seem unavoidable, resulting from basic human responses to our perceptions of others’ behaviour, and subsequent judgements of the appropriateness of praise or blame. We can moderate such responses according to mitigating factors, but it is difficult to see how we could, and why we should, do away with them altogether.

As well as the unavoidability of responsibility judgements, holding people responsible can also help to ensure efficiency and fair allocation. ‘Moral hazard’ occurs when the costs of an agent’s actions are spread among a larger group of individuals, while the benefits accrue to him or her alone, encouraging self-interested behaviour (think of the dining companion who orders the steak and most expensive wine before insisting that the bill be split evenly among the table). Moral hazard can arise where there is no opportunity to hold people responsible and can create a ‘tragedy of the commons’. Ensuring that people directly bear the costs of their behaviour through holding them responsible can encourage collectively advantageous and prudent behaviour and discourage short termism and recklessness.

Given these features, it is not surprising that references to responsibility appear in policy language, healthcare practice and media reports relating to health. First, it arises frequently in policy language, often framed as facilitating patient empowerment and the need to respect individual choice. For instance, the National Health Service (NHS) Constitution for England states:

The NHS belongs to all of us. There are things that we can all do for ourselves and for one another to help it work effectively, and to ensure resources are used responsibly… [including recognising] that you can make a significant contribution to your own, and your family’s, good health and well being, and take personal responsibility for it.^8^

The current UK Health and Social Care Secretary, Matt Hancock, gave a speech in 2018 in which he repeatedly emphasised the need for individual responsibility to play a role in public health and preventative approaches to healthcare:

I think we need to pay more attention to our responsibilities, as well as our rights.

Today, I want to talk about those responsibilities, and our task for the National Health Service to help empower people to take more care of their own health…

I want to see people taking greater personal responsibility for managing their own health. For looking after themselves better, so staying active and stopping smoking.

Now, I want to address head on how we can do this without undermining people’s liberty…

Because focusing on the responsibilities of patients shouldn’t be about penalising people but about helping people to make better choices…

[b]y giving people the knowledge, skills and confidence to take responsibility for their own health.^9^

Second, the view that health promotion is achieved by encouraging people to take responsibility for their own health is apparent in social marketing tools used to inform and educate people about healthy lifestyles (see, for instance, the NHS Livewell website).^10^ If an approach to health promotion assumes that people can and should take responsibility for their health—specifically when it comes to ‘avoidable’ conditions that result from lifestyles (eg, smoking-related and obesity-related disease)—one might expect healthcare resource allocation decisions to disadvantage groups seen to have failed to take responsibility in this way. There are some illustrative cases within the NHS: for instance, a number of clinical commissioning groups in England and Wales require those who smoke or have a body mass index over 30 quit smoking/lose weight before they are referred for elective surgeries.^11^ Failure to meet these demands can result in longer waits to access surgical treatment or denial of treatment altogether. This is despite a lack of evidence that smoking/obesity reduces the therapeutic benefit of those surgeries.^12^

Finally, responsibility also features prominently in narratives around health behaviour in the mainstream press, which often condemns recklessness relating to health, particularly where this causes third-party harms.^13 14^ For instance, heavy criticism is directed at pregnant women who smoke, drink alcohol or engage in activities deemed ‘risky’ to the fetus.^15 16^ These ‘bad behaviours’, along with smoking, obesity, failure to vaccinate one’s children or attend screening programmes, attract negative stereotypes and can become heavily stigmatised.^17–19^ Ethicists have also directly called for the stigmatisation of unhealthy behaviours. For example, Callahan has argued in defence of the stigmatisation of obesity, arguing that it is both unavoidable and justified in pursuing health gains.^20^ Singer has argued that those who ‘weigh more’ should ‘pay more’ when flying, both as an incentive to encourage weight loss and as an issue of fairness.^21^

These discussions propose that people should avoid unhealthy behaviours and/or bear the costs of their actions and/or are fairly subject to moral criticism on the basis of their actions. Such propositions seem to assume that people are morally responsible for those actions and perhaps for any resultant health harms. Yet, this obscures the extent to which an individual’s health is often the product of repeated behaviours over time and is heavily influenced by other agents. Since much of the discussion around responsibility and health is concerned with the impact of habitual (‘lifestyle’) behaviours on the development of chronic disease (such as heart disease, various forms of cancer and type II diabetes), it is particularly important to consider the diachronic and dyadic (terms we use to recognise responsibility-over-time and responsibility-across-persons) aspects of responsibility.

We do not commit to a defined set of necessary and sufficient conditions for responsibility here since our account is consistent with different approaches to specifying such conditions. A rough idea will be useful, however: what we mean when we describe someone as (morally) responsible for some action is that they fulfil some version of the epistemic and control conditions for responsibility.^22–24^ Thus, agents must have sufficient awareness of the likely consequences of their actions—including an understanding of their moral significance—along with sufficient control over their actions in order to be considered morally responsible for those actions. We also assume that the kind of responsibility of interest is merit based, as opposed to consequentialist, and that we are not allocating responsibility merely to ensure efficiency but to track something deeper. It is important to note that moral responsibility can come apart from the appropriate allocation of things like praise and blame, at least on some accounts.^25^ We stop short of arguing that any *particular* response will be justified on the basis of responsibility for some action and so we remain neutral as to the tightness of the relationship between responsibility and blame.

A note on actions and outcomes: our aim is to indicate how an account of moral responsibility might better reflect the reality of the relationships between agents, health-related behaviour and diseases. Any appetite to incorporate responsibility practices into health policy and practice is likely motivated by a desire to make healthcare more efficient, that is, to improve health outcomes. Thus, one might assume that discussion of responsibility in this context is concerned with responsibility *for health outcomes*. We focus, however, on health-related behaviour (such as smoking, diet, physical activity and so on) and consider how agents are responsible for these actions, rather than the diseases that such actions make more likely. We assume that, if agents are responsible for health harms they suffer, this will be due to their antecedent responsibility for some action that was causally implicated in that harm coming about. Thus, responsibility for the health-related behaviour will be necessary (though not sufficient) for responsibility for the resulting health outcome. While the latter might ultimately be of interest, here, we focus our discussion on the former.

Often, it is thought that allocations of praise or blame will follow from the identification of moral responsibility. Elsewhere we have argued that the kinds of reasons people have to be healthy are not always moral, and so failure to act according to them does not result in a moral failure or make one morally blameworthy.^26^ Here we are not only concerned with moral responsibility insofar as it warrants praise or blame, but more broadly, insofar as it identifies actions and consequences that can be attributed to an agent.^i^ We thus assume that one may be morally responsible for becoming obese through overeating without assuming that any negative (or indeed positive) assessment of that behaviour is therefore justified, much less that it warrants public praise/blame and reward/punishment. Effectively, one can be morally responsible for something that is morally neutral.

The significance of this is that attributions of desert are often thought to be justified on the basis of moral responsibility, even when the product of the morally responsible action is morally neutral. Desert guides how we think about the appropriateness of allocating goods. For instance, if Sam makes himself a cup of tea, we think it should be Sam that gets to drink it. The making of the tea—the planning, the effort exerted and so on—acts as a desert base for Sam getting to drink the tea: Sam *deserves* to drink the tea. In fact, even without invoking desert, we tend to think that the consequences of events should connect to the agent whose actions constitute those events; this can also include further downstream consequences, though how much further downstream and which consequences is contentious.^3^ Imagine a magical hangover-transferring machine: if you enter the machine before going to sleep after a heavy night’s drinking, your hangover will be miraculously transferred to another person. This seems troubling: without claiming that you *deserve* a hangover as a result of your heavy drinking (it would be fine if you got away scot free and awoke perfectly refreshed), it seems that if a hangover is to be had, it is you that should have it.

The relevance of this to health is that some want to claim that responsibility-sensitive health policies are justified on the basis of the intuitive judgement that actions should link to consequences.^1 27^ Such a claim does not require that one deserves *additional* punishment for one’s unhealthy behaviour, but that one’s moral responsibility for that behaviour makes it appropriate that one bear the costs (or benefits) that arise as a result. For the purposes of our discussion, we assume that the intuition that moral responsibility has presumptive relevance cannot be dismissed out of hand, and we seek to explore below how an account of responsibility might better reflect the temporal and multipersonal dimensions of responsibility in health.

## Challenges to responsibility in healthcare

To recap, incorporating responsibility practices into healthcare may be argued to be parsimonious, intuitive and effective. First, since responsibility practices operate across many domains of life, it might seem obvious that they ought to operate in the healthcare context as well. Second, identifying agents as responsible for certain actions is a highly intuitive practice and people frequently think and act in terms that assume agents are either morally responsible for some action or attitude or can be excused from moral responsibility for some reason: one way or another, identification of responsibility is often implicit in social reasoning. Finally, attending to moral responsibility may be assumed to ensure efficiency by avoiding the problems of moral hazard and regulating people’s behaviour in ways that benefit society. If effective, this would save healthcare systems money while improving population health. Further arguments can be—and have been—put forward that rely on claims that holding responsible is in-and-of-itself a good thing, or that it is good for people to get what is deserved, or that all else being equal it is preferable to prioritise treating those who bear no responsibility for their illness over those who do (as luck egalitarians such as Segall would maintain).^27^

There are, however, challenges to the inclusion of responsibility practices in healthcare. These relate to difficulties in practical implementation in particular health contexts, ethical concerns about the effects of holding people responsible, as well as philosophical objections to the idea that people are morally responsible for their health in meaningful ways.^3–5 28 29^

We do not seek to adjudicate between these debates here but note that some of these arguments provide compelling reasons both for and against a role for responsibility practices in healthcare. We turn instead to discuss how classic conceptions of responsibility are ill-adapted to guide action in areas of healthcare where it is often thought to be important, particularly the context of ‘lifestyle-related disease’. This informal term typically refers to chronic diseases that are linked to exposure to behavioural risk factors such as smoking, drinking alcohol, physical inactivity and poor diet. Chronic disease related to these risk factors contributes significantly to global mortality and healthcare expenditure and is a priority area for public health systems.^30^ We thus argue that, *if* responsibility practices are to have a role in healthcare, some revision to the concept may be required in order for it to be meaningfully applied in these contexts.

## Responsibility over time and across agents

Habitual, health-related behaviour presents a challenge to accounts of responsibility. On the one hand, it seems absurd to claim that individuals are not responsible for routine, everyday behaviours that they repeatedly engage in over long periods. Yet, on the other hand, many individuals struggle to exert control over these behaviours. This results in people engaging in behaviours they know could be harmful, often despite wishing to alter those behaviours.^31 32^ One might be sympathetic to the views of both those who argue that *of course* people are responsible for their behaviour, as well as those who argue that surely the apparent lack of control indicates precisely the opposite.

We think this debate is of limited application. We propose that discussions of responsibility in the context of health-related behaviour must pay more attention to the *diachronic* and *dyadic* aspects of responsibility. These reflect how a person’s responsibility for her health is something that is exercised over time and something that can be shared between members of small groups. For the purposes of simplicity, we will consider only one’s partner or close other (spouse, de facto partner and so on). Thus we call this aspect dyadic. While existing discussions of responsibility do sometimes refer to these temporal and interpersonal aspects of responsibility, they rarely receive explicit attention in discussions of the practical applications of responsibility (for instance, in health). An exception is the interest in describing accounts of collective and social responsibility, particularly in the context of collective action problems, and some recent contributions that argue in favour of ‘socialising’ responsibility.^33 34^

In the following sections, we sketch out how the diachronic and dyadic dimensions of responsibility are relevant to considerations of people’s responsibility for their health-related behaviour.

## Diachronic responsibility: responsibility over time

While some health behaviours are ‘one shot’, such as long-lasting vaccinations, sterilisations and surgeries, others have to be repeated frequently over time to affect health: brushing one’s teeth daily and routinely taking antiretroviral drugs. This is true of health harming behaviour as well as behaviour that promotes health: breaking a leg in a skiing accident or overdosing on heroin can have immediately devastating effects on health, whereas regular lack of exercise, overeating, smoking and excessive use of alcohol are associated with the cumulative increase in risk of harm to health.

Making global judgements about an individual’s responsibility for his or her health will require making a judgement about his or her responsibility for those behaviours that contributed to her health (or lack of it). This requires a way of judging responsibility for behaviours repeated over time, where no single instance of that behaviour can be considered health harming/promoting to any meaningful extent. Take an individual who smokes. It seems misleading to say he or she *harms* his or her health by smoking a cigarette, since the tiny increased risk of diseases such as cancer and heart disease that he or she experiences as a result of smoking a single cigarette seem too marginal to be considered harmful. Furthermore, the minimal increase in risk of harm a single cigarette creates might be outweighed by the pleasure the individual experiences in smoking it, depending on his or her personalised risk/pleasure profile. Thus, while we might make a judgement about the responsibility he or she holds for the behaviour of smoking a single cigarette, this responsibility is distant from any responsibility that relates to the development of heart disease down the line.

If we are ultimately interested in making judgements about responsibility for health related disease, the responsibility an individual bears for a single instance of a behaviour that in all likelihood has no impact on his or her health will not be very interesting. Instead, we must direct our consideration of the smokers’ behaviour to the responsibility he or she bears for the *repeated behaviour* of smoking cigarettes over many years, since this is what is likely to significantly contribute to his or her risk of developing heart disease.^ii^ It is worth noting that someone who is judged diachronically responsible for smoking need not, ultimately, be judged responsible for any consequences that result from his or her smoking (including health harms). However, we assume that a necessary condition of responsibility for such consequences will be that he or she was diachronically responsible for smoking.

Judging diachronic responsibility is a more complicated task with a number of options available for making the assessment. We might demand that *every* instance of the relevant behaviour fulfils the conditions for responsibility, or demand that the *majority* of instances of the behaviour meet the conditions for responsibility, or that a minimum *threshold* number of instances do so and so on. Depending on which of these is selected, the demands for identifying responsibility will be more or less stringent.

Whether diachronic responsibility is met with regards to smoking will depend on what conditions are set for responsibility and how these are met over time.^iii^ So, if we use the epistemic and control conditions as a means of identifying responsibility, we must further consider how these conditions are to be met across time. The regularity with which the epistemic and control conditions must be met to satisfy diachronic responsibility may be called the *diachronic condition*. All of the epistemic, control and diachronic conditions may be more or less demanding, as illustrated in table 1.

**Table 1:** How the epistemic, control and diachronic conditions can vary along a spectrum of demandingness

Condition	Level of demandingness		
	High	Moderate	Low
Epistemic	Extensive knowledge about consequences of action, including their moral significance, and how to bring about/avoid particular consequences.	Some knowledge about consequences of action, though potential for misunderstandings/failure to integrate into one’s own circumstances.	Little knowledge about consequences of action and how it is relevant to one’s behavioural decisions/fulfilment of projects/moral obligations.
Control	Total control over one’s behaviour, enacted with ease.	General control over one’s behaviour though some struggle may be required and occasional failure to enact behaviour as intended.	Poor control over behaviour.
Diachronic	Epistemic and control conditions met on all instances of behaviour.	Epistemic and control conditions met on many/some instances of behaviour.	Epistemic and control conditions rarely/never met regarding particular behaviour.

We can rate the extent to which the epistemic and control conditions are reached diachronically for our smoker. If he or she is reliably well informed about the likely effects of his or her smoking, and on every occasion he or she smokes a cigarette, he or she is aware of the potential harm of her behaviour, then he or she will fulfil even demanding epistemic and diachronic conditions. If, however, his or her capacity to control his or her behaviour fluctuates, and he or she is only sometimes able to refrain from smoking, then he or she will only meet the control condition for responsibility if the diachronic condition is weakened.

There is, thus, an interaction between the epistemic, control and diachronic conditions: the more demanding the epistemic and control conditions, the less likely someone will be to fulfil the diachronic condition. Likewise, if the diachronic condition is very demanding, one is less likely to fulfil the epistemic and control conditions. It is also common to speak of responsibility as a scalar concept, whereby one may be more or less responsible for some action. In this case, one could also assess the degree to which an agent meets the epistemic, control and diachronic conditions at different levels of demandingness.

There is a second sense in which responsibility can be diachronic: an agent can take actions today that will affect how he or she is able to act tomorrow, or next week or in a year’s time. Particularly when it comes to hard-to-change behaviours, agents might take steps to alter the environments their future selves will inhabit in order to make changing those behaviours easier. In the clinical context, upstream decision making can influence treatment options later available to patients and carers.^35^ Engaging in commitment contracts, such as throwing out alcohol or paying for a year’s gym membership, enables a person at one time to influence her future behaviour.

The assessment of diachronic responsibility will thus also require the adoption of a position on the continuity of personal identity over time. Assuming perfect continuity of personal identity over time: if a person *P*, at time t acts in such a way as to make it likely that he or she will smoke a cigarette at some point in the future (t+n), so long as *P*_t_ fulfilled the epistemic and control conditions for responsibility at t with regard to his or her behaviour at t+n (ie, she was aware of the likely consequences of her behaviour and was able to control her behaviour, to the degree necessary to meet the demandingness of those conditions) then *P*_t_ will be (prospectively) responsible for *P*_t+n_’s behaviour, even if he or she does not appear to meet the conditions for responsibility at *P*_t+n_. Similarly, *P*_t+n_ will be retrospectively responsible for *P*_t_’s behaviour. This equates roughly to the ‘tracing’ condition of responsibility and has been much discussed in the related responsibility literature.^36^

However, for behaviours that take place across lifetimes—many smokers start in adolescence and experience the most severe ill effects around retirement—it may be too quick to assume that personal identity will be perfectly conserved. This presents the possibility that *P*_t+n_ is not ‘fully’ retrospectively responsible for the past behaviour of *P*_t_, even if fully responsible at t, and *P*_t_ should not be considered ‘fully’ prospectively responsible for the future behaviour of *P*_t+n_. The scare quotes around ‘fully’ indicate that there will be some indeterminate weakening in the extent to which *P* is responsible, since it seems the extent to which her responsibility is diminished will depend on details about the accepted account of how personal identity varies over time, the extent to which the consequences of the agent’s actions were genuinely foreseeable and how this relates to responsibility.

Depending on the position on continuity of identity adopted, this aspect of diachronic responsibility might be modelled differently. One might wish to maintain that *P*_t_ and *P*_t+n_ *are the same* with minimal or no weakening of identity or responsibility over time. Alternatively, one might model *P*_t_ and *P*_t+n_ as non-identical, perhaps fully distinct agents. In this case, *P*_t_ may only bear responsibility for the actions of *P*_t+n_ in so far as one agent can be responsible for influencing the behaviour of another. If the latter option is selected, then diachronic responsibility may depend on an account of dyadic responsibility—responsibility distributed across agents—which we introduce in the next section.

This has implications for those whose past actions give them a poor choice set, pushing them to act imprudently. In particular, this is of relevance to habitual behaviours, such as smoking, drinking, diet and physical activity, since established habits are difficult to change: they are less flexible, less subject to conscious deliberative control and they are cued by environmental stimuli. Some unhealthy habits (such as smoking) can also be addictive, making them even harder to discard.^37^ Others, such as overeating or lack of physical activity, might fall short of addiction yet nonetheless involve ways in which an agent’s capacity to control his or her behaviour is substantially weakened by his or her previous behaviour (some argue that behaviours such as overeating may be even more challenging to control than classic substance addictions, see ref 38).

Even so, golden opportunities may arise, whereby agents at a particular point in time have ample opportunity to alter their behaviour.^6^ Agents who previously did not meet the conditions of responsibility but subsequently fail to capitalise on golden opportunities may well ultimately be considered diachronically responsible, if they can be made aware of the golden opportunity and its importance. Since this may be enabled through other agents, this can indicate another instance where dyadic and diachronic responsibility relate to one another.

We should not adopt a position on the appropriate account of personal identity here, nor how it should influence diachronic responsibility. It is worth noting, however, that attending directly to the diachronic nature of responsibility for habitual, health-related behaviour will require a commitment to some view of personal identity: the extent to which it is continuous over long periods of time, in what situations it becomes discontinuous and the ways in which identity interacts with responsibility.

## Dyadic responsibility: responsibility *across persons*

Agents’ behaviour is influenced by the environment, and part of that environment will be other agents. In particular, partners, friends and colleagues with whom one cohabits, socialises and/or spends long periods of time with will shape one’s behaviour through their presence and actions. This will apply to health-related behaviours such as smoking, eating, exercising and drinking.^39^ In this section, we seek to show that the involvement of a person’s partner in health-related behaviours has relevance for how we conceptualise responsibility for those behaviours.

Agents are subject to moral evaluation in a way that non-agents are not, so we might evaluate agents’ actions (and their impact on other agents) in ways that would not apply to non-agents (dogs, cars, beer and weather). To the extent a person fulfils the epistemic and control conditions, he or she will qualify as responsible for his or her behaviour, including the impact it (foreseeably and avoidably) has on other agents. For instance, offering one’s partner a cigarette, despite knowing he or she is trying to quit and bringing sweet snacks into the home and increasing the calories consumed by one’s spouse: an agent might be morally responsible for these behaviours and be subject to moral evaluation on the basis of the benefits/harms they cause to their loved one.

One might assert that this is consistent with individualistic accounts of responsibility for actions. A person is responsible themselves for choosing to smoke a cigarette that has been offered or eating sweets in the pantry. It is not the spouse who is responsible. However, this views control as all or none, whereas it is patently variable. People can exert more self-control when not tired, distracted, intoxicated and so on.^40^ One’s partner can play an important role in buttressing strength of will in relation to such circumstances. Moreover, one owes one’s partner a duty to support them in achieving their goals, developing as a person and achieving a good life. Part of being in a close personal relationship is sharing one’s life and being a part of a ‘team’. Ethically, this requires supporting, not undermining or being neutral, to one’s partner’s health-related goals and taking actions consistent with this.

Increasingly, social scientists are also looking at the role of groups, particularly small groups such as intimate dyads, in order to understand the processes governing health-related behaviour.^41–43^ Research into habitual, health-related behaviours and chronic disease management is finding interventions targeting dyads could be more fruitful than those targeting individuals alone.^44 45^ For instance, a common phenomenon of study in social/health psychology is *self-efficacy*. Self-efficacy is the degree of confidence a person has that he or she is capable of carrying out some behaviour or achieving some end (eg, quitting smoking) and is thought to influence success in behaviour change (ie, higher self-efficacy results in higher rates of successful behaviour change).^46^ This concept can be expanded to the level of the group, becoming *collective or dyadic efficacy—*the degree to which a collective or dyad has confidence in its capacity to effect change.^47^ This indicates new areas for research and potential points of intervention.^48 49^

Within philosophy, there has also been appetite for extending the unit of analysis beyond the individual. Influential to our discussion has been recent work in the philosophy of psychology by Richard Holton.^50^ Holton articulates the idea that an individual’s memory can be ‘buttressed’ by close others with whom they have deep social relationships. This is important in cases where pathologies (such as dementia) interfere with memory retrieval, since the engagement of other people can facilitate remembering. Memory is often thought to have an important role in identity, thus it looks like the actions of others, in facilitating remembering, can act to preserve a person’s identity.^51 52^ Along similar lines, the psychologist Daniel Wegner proposes that dyads can have a ‘transactive memory’, whereby one agent ‘knows’ things that are stored in the brain of the other agent.^50^ Such ways of framing cognition are consistent with Clark and Chalmers’^53^ proposed theory of the extended mind, whereby the ability to store and readily retrieve information from sources external to the brain is taken to show that the limits of the mind ought not to be set at the edges of the skin.

In discussing responsibility directly, Levy^54^ has recently appealed for philosophers to recognise their error in focusing (almost) solely on the individual (as bounded by skin) as the locus of agency and recognise that agency—and responsibility—is invariably ‘socialised’. While we agree with much of what Levy says, we think it is mistaken to jettison entirely the notion of the individual as bounded by the skin—the physical distinction between our bodies and the outside world often has an entirely unambiguous significance. Furthermore, we think there are relevant differences between influences on an individual that emerge within the context of a special relationship; those which come from agents who are strangers to the individual; and those which emerge from the natural environment. Both agency and relationships matter in the context of influence and responsibility. This has been explored in the healthcare context in relation to the important role played by families in the context of decision making about treatment and provision of ongoing care.[Verkerk *et al* 2015; Lindemann *et al* forthcoming].

If it is plausible to think that cognitive systems such as memory and identity can be importantly distributed between agents, then the same may also be true of responsibility. In addition, individuals may not necessarily be the most useful or parsimonious unit of analysis in the context of health-related behaviours due to the profound effects of social life and close relationships.

There already exists a discussion of responsibility at the large group level in the literature on collective responsibility. Collective responsibility attaches to a particular group of agents in relation to some consequence. It often refers to actions that must be undertaken by numerous individuals in cooperation in order to achieve a particular effect (for instance, reducing carbon emissions to avert climate change). Some accounts of collective responsibility go beyond the cumulative responsibility of individual agents within a group to propose that it is the group itself that holds the collective responsibility. This may or may not involve identifying a distinct entity—the group agent—to which collective responsibility attaches.^55^

What we here call dyadic responsibility is a small-scale species of collective responsibility. Our proposal is: many health-related behaviours that result in health gains/losses to a particular individual are the product of the actions or inactions of numerous agents. In some circumstances—intimate dyadic relationships, for instance—agents have significant impact on one anothers’ behaviour. Moreover, within the context of those relationships, there are particular expectations of concern for and even obligations to anothers’ well-being, obligations to act to support one another’s interests, respect for each others’ preferences and projects, shared life goals, mutual dependence and so on. As such, the responsibility of one member of the dyad may be ‘outsourced’ to the other member. This we call ‘dyadic responsibility’.

To illustrate, consider Jack and Jill, a romantic couple. Jill is trying to lose weight. Since Jack does the majority of the cooking, Jill relies on him to cook food that is low in calories in order to facilitate her weight loss. While Jill is ultimately the agent who lifts the food to her mouth and eats it, Jack plays an important role in what she eats. Consider now Joe. Joe is a chef at Jill’s work cafeteria. Joe also has a significant impact on what Jill eats, but his obligations towards Jill are much weaker (for sure, he should avoid poisoning her, but he knows nothing of her goal to lose weight, nor is it clear that he should care, much less change his behaviour, if he did know). Jack shares in and supports the goals of Jill in a way that Joe does not. While Joe bears run of the mill, individual responsibility for not poisoning Jill with the food he cooks, in this case, unlike Jack, Joe is not a member of a dyad to which dyadic responsibility attaches.

Dyadic responsibility picks out where one agent plays a key constitutive role in the responsibility of another agent for actions he or she takes. It emphasises the duties and obligations between those who sit in close relationships with one another, and how that should be reflected in their obligations with regards to how they influence one another’s behaviour. Ultimately, dyadic responsibility will affect how responsibility for outcomes will be shared between agents. This could have interesting implications when considered in the light of debates about responsibility for health. First, it provides a way of maintaining some room for responsibility practices to play a role in discussions of health-related behaviour that emphasise the importance of social and environmental influence on behaviour (and tend to play down the role of pure individual agency). Dyadic or collective responsibility is compatible with situations where one individual’s behaviour is heavily influenced by other agents. To the extent to which this is often the case (ie, individuals often exist in close relationships to others’ with each agent’s behaviour impacting on the other’s), dyadic responsibility may be an important variety of responsibility that is missing from current discussions, and insofar as this is the case, giving a name to dyadic responsibility may be a first step towards developing strategies for responding to, or making use of, this phenomenon. This could include adapting practices of holding responsible to appropriately respond to dyadic responsibility, or finding ways of supporting dyadic responsibility (such as targeting healthy eating interventions at dyads, for instance).

## Concluding remarks: implications of diachronic and dyadic responsibility

While the appropriate role for responsibility practices in healthcare is disputed, responsibility thinking might be an inevitable—and not wholly unwelcome —feature of human psychology and social behaviour. In any event, it seems that assumptions about responsibility and an ambition to recognise people’s responsibility for their health is sometimes integrated into health policy and practice. It is thus unclear that we either could nor should seek to exclude responsibility practices from discussions of health, including in the context of lifestyle-related, habitual behaviours that contribute to the development of chronic disease, as well as the management of chronic and long-term conditions once they develop. However, we have argued here, if we are to incorporate responsibility into healthcare, we must make some alterations to accounts of responsibility in order to better recognise the ways in which behaviour occurs *over time* and *across people*.

We have labelled these dimensions of responsibility *diachronic* and *dyadic* responsibility and sought to provide an initial sketch of what they could look like: what features of responsible actions they would need to pick out and what information about the world/agents they could better reflect.

Diachronic responsibility specifies an additional, diachronic condition for responsibility, which describes how the other conditions for responsibility need to be met over time in order for an agent to be considered responsible for his or her behaviour. This helps us recognise how the conditions of responsibility may vary over time and to address head on questions of how personal identity is continuous over time and interacts with responsibility. It also recognises that even under long periods of diminished control, if an agent has had periods of opportunities to exercise diachronic responsibility, he or she can still be held responsible.^6^

Dyadic responsibility recognises the role close others can play in an agent’s behaviour and proposes that this should be incorporated into assessments of responsibility. We focus here on intimate dyads and relationships where special obligations, shared goals and so on create a basis for thinking that one agent’s role in another’s health could carry more meaning than mere causal impact. This is the beginning of a project to take fuller account of social relationships into a theory of responsibility. In developing this further, we hope to provide a communitarian account of responsibility: one that recognises that people’s behaviour, values, projects and efforts are deeply wrapped up in social relationships. For now, considering the responsibility of dyads may have practical uses: reflecting opportunities for intervention to promote health, both by adopting habits that reduce the likelihood of developing chronic disease and by facilitating adherence with medically advised approaches to managing long-term conditions. Considering responsibility at the dyadic level may also be more consistent with empirical models of agency. We propose that diachronic and dyadic responsibility can be combined with different accounts of responsibility. Although we have used the language of epistemic and control conditions here, these can be explicated in different ways, and altogether alternative approaches may also be used.

Alongside the inclusion of responsibility into overarching health policy framings, the real ‘bite’ of responsibility is likely to arise in its translation into practical contexts. One area where the appropriate allocation of responsibility could be particularly important is in relation to the management of long-term and chronic conditions, such as diabetes, hypertension, depression, HIV and many more pathologies that benefit from active patient engagement with self-management strategies, adherence to treatment plans, shared decision-making practices and the involvement of supportive social networks. The ongoing nature of such conditions makes the dyadic dimension particularly important here and provides interesting opportunities for investigating how the dyadic and diachronic aspects of responsibility interact (for instance, as previously mentioned, the way in which partners may highlight golden opportunities to adopt health-promoting behaviours). In applying notions of responsibility to such contexts, it will be vital to carefully integrate them with appropriate understandings of what will be in the patients’ (and others’) interests and what will constitute suitable therapeutic goals and strategies.^35 56^

### Recognising responsibility

Diachronic and dyadic responsibility draw attention to factors (time and agents) that are relevant to identifying who is responsible for what and to what extent. This allows a more fine-grained analysis of responsibility that better reflects the role agents play in their behaviour, which will assist in making further determinations about what should follow from responsibility ascriptions (in terms of praise, blame, personal relationships, access to social goods and so on). If appropriately recognising responsibility is a requirement for respect, then ensuring the diachronic and dyadic dimensions of responsibility are accounted for will be supportive of more accurate and more respectful modelling of agents.

At the practical level, there will be difficulties balancing appropriate identifications of responsibility with intuitive responses to perceived responsibility. People’s (including healthcare professionals’) identification of responsibility may come apart from when patients *in fact* have the capacity for responsibility (eg, are able to recognise relevant reasons and change their behaviour in line with treatment plans, engage in shared decision making and so on). These judgements may be affected by factors such as affective responses and personal preferences, particularly in patients with conditions that result in antisocial behaviour. Any value of ‘recognising responsibility’ may be dependent on *successfully* and *appropriately* recognising responsibility and the extent to which it is present, without introducing inappropriate and potentially harmful responsibility practices.^57^

### Enhancing responsibility

If (and we have not discussed this here) responsible agency is a good thing, it may be desirable to seek to promote responsible action. Diachronic responsibility indicates how this could be done by encouraging agents to take steps that will enhance their future responsibility for their actions, such as through the use of self-binding ‘commitment’ contracts, longer term behaviour change programmes, support groups and so on. Temporal distance may result in people identifying less with their future selves and engaging in excessive future discounting about benefits and harms. Thus, encouraging a sense of connectedness with one’s future self could also help people to take steps to promote their own diachronic responsibility (there is some evidence from psychology to support this).^58–60^ Recognising that responsibility can be dyadic also indicates opportunities for responsibility enhancement by targeting not just the subjects of responsibility themselves but those with whom they share close relationships and who are able to contribute to (or detract from) their responsibility.

### Holding responsible

As discussed, there is appetite within health systems to encourage people to ‘take responsibility’ for their health, particularly regarding the contribution of habitual behaviours to chronic disease. Diachronic responsibility suggests we must be careful in assuming that observable, occurrent, responsible action reflects longer term responsibility for poor health outcomes. Alternatively, an evaluation of diachronic responsibility that concluded an agent was regularly responsible for his or her behaviour (say, smoking) might indicate a much stronger case for assigning responsibility. So too if the agent had, over time, been presented with significant golden opportunities to take diachronic responsibility. Factors other than responsibility must be considered when engaging in practices of ‘holding responsible’, which ordinarily include the attachment of blame, punishment, stigma and so on.^61^ However, establishing responsibility will almost always be a necessary first step.

Dyadic responsibility also has potentially revisionary implications for practices of holding responsible. To the extent that intimate dyads can play a central role in another’s actions and share in their responsibility, they could also be appropriate subjects of the consequences of holding responsible, including praise or blame. Clearly, this will need to reflect the actual extent to which one is able to influence another’s behaviour, as well as the degree to which such interference would be appropriate. However, in principle, attributing retrospective responsibility to members of dyads could be as justifiable as attributing it to the individual alone.

As discussed elsewhere, the non-ideal circumstances presented by healthcare contexts make them difficult instances for, first, evaluating responsibility, and second, enacting practices of responsibility such as ‘holding responsible’.^62^ Other factors—beyond the fulfilment of the epistemic and control conditions (and consideration of their diachronic and dyadic aspects)—will need to be considered in determining how responsibility for particular actions can and should be translated into practices of holding responsible, such as prioritising certain people for treatment above others.

We think that some disagreements regarding people’s responsibility for their health-related behaviour may derive from failing to take due consideration of the diachronic and dyadic aspects of responsibility. Attempts to explicitly address how the conditions of responsibility must be met over time, and the ways in which responsibility is appropriately shared within personal relationships may help better address these disputes.

## Footnotes

i A number of philosophical accounts of responsibility make room for the possibility of responsibility in the absence of blameworthiness, praiseworthiness and so on. Watson, for instance, distinguishes between *attributability* and *accountability*, with the later justifying social censure but not the former; Scanlon provides an account focused on the quality of interpersonal relationships and explores how moral responsibility may come apart from appropriate practices of blaming and other responses.^63 64^

ii Some might find it appealing to characterise the ‘action’ of smoking as the repeated behaviour over time, rather than (as we have) as numerous distinct instances of action. The individuation of acts is rather complex, and we do not think it makes a significant difference to our account, here, if the action is taken to be repeated or is taken to include all the repetitions, since on either approach it seems that responsibility must be judged diachronically.

iii See also Levy’s instructive account of how the autonomy of unwilling drug addicts may be revealed to be undermined due to preference oscillation over time.^65^ There is not room to discuss here how responsibility and autonomy connect, but it seems that significantly impaired autonomy could be grounds for absolving one from responsibility.
